# Preparation and properties of room temperature vulcanized silicone rubber based on rosin-grafted polydimethylsiloxane†

**DOI:** 10.1039/c7ra13672b

**Published:** 2018-04-19

**Authors:** Qiaoguang Li, Xujuan Huang, He Liu, Shibin Shang, Zhanqian Song, Jie Song

**Affiliations:** Institute of Chemical Industry of Forestry Products, Chinese Academy of Forestry, Key Laboratory of Biomass Energy and Material, National Engineering Laboratory for Biomass Chemical Utilization, Key and Laboratory on Forest Chemical Engineering, State Forestry Administration Nanjing 210042 Jiangsu Province China liuheicifp@caf.ac.cn +86-25-85482499 +86-25-85482452; Institute of New Technology of Forestry, Chinese Academy of Forestry Beijing 100091 China shangsb@hotmail.com songzq@hotmail.com; Department of Chemistry and Biochemistry, University of Michigan-Flint Flint Michigan 48502 USA

## Abstract

Rosin-grafted polydimethylsiloxane (RGSO) was prepared *via* ring-opening reaction of glycidyl ester of rosin acid (ER) with hydroxy-terminated amino polydimethylsiloxane (PDMS). The structure of RGSO was confirmed by ^1^H and ^13^C NMR spectroscopy. The effects of ER on relative molecular weight and rheological properties of RGSO were studied by gel permeation chromatography and rotational rheometry. Then, room temperature vulcanized (RTV) silicone rubber modified with rosin was prepared using RGSO, hydroxy-terminated PDMS, tetraethoxysilane, and organotin catalyst. The structures and properties of RTV silicone rubbers were studied by scanning electron microscopy, thermogravimetric analysis, a universal testing machine and dynamic mechanical analysis. The rosin-modified silicone rubber showed remarkably improved thermal and mechanical properties. Temperatures corresponding to 10% weight loss and maximum rate of weight loss increased by 66 °C and 177 °C, respectively. Moreover, the tensile strength and elongation at break increased by 138% and 113%. The role of rosin structure in improvement of properties is discussed.

## Abstract 2

Rosin-grafted polydimethylsiloxane (RGSO) was prepared *via* ring-opening reaction of glycidyl ester of rosin acid (ER) with hydroxy-terminated amino polydimethylsiloxane (PDMS).

## Graphical abstract

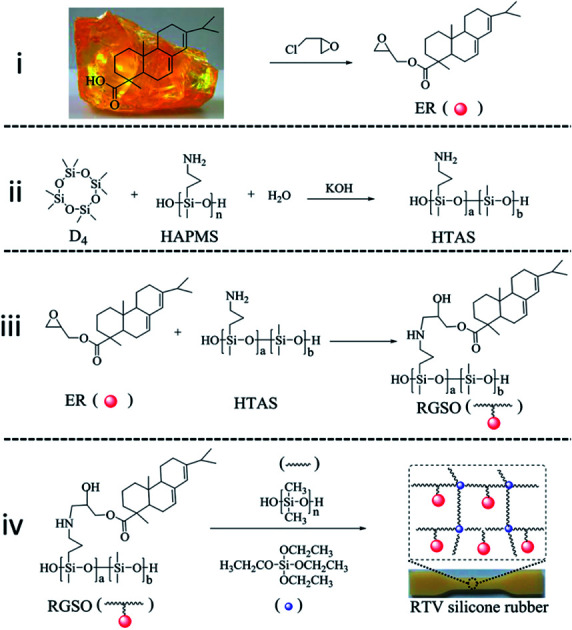

## Introduction

Silicone rubber has gained a lot of attention, due to its unique physical and chemical properties and its special structure, consisting of alternating Si–O backbone chains with organic side chains.^1^ The unique physical and chemical properties of silicone rubber include: remarkable heat resistance, ozone resistance, weather resistance, *etc.*^2^ Because of these merits, silicone rubber has important roles in the construction, healthcare, and electronics industries.^3^ However, the relatively low mechanical strength of silicone rubber, due to low cohesion energy density, affects its practical applications.^4,5^ Physical or chemical modification is carried out by adding fillers or hard phases in order to improve the mechanical properties of silicone rubber.^6^

Chemical modification, such as copolymerization, crosslinking, or grafting, is an effective way to significantly alter, optimize, and improve the properties of polymers.^1^ Liu *et al.* suggested that grafting of room temperature vulcanized (RTV) silicone rubber with polyhedral oligomeric silsesquioxanes (POSS) to obtain RTV silicone rubber-*g*-POSS can significantly enhance the thermal stability, because of chemical incorporation of POSS into polydimethylsiloxane (PDMS) chains.^7^ Meng *et al.* reported improvement in the thermal stability of poly(methylvinylsiloxane) and found that the grafted POSS inhibited the rate of thermal degradation of silicone rubber.^8^ Not only POSS with their combined inorganic–organic properties, but also rigid cyclic aliphatic or aromatic petrochemical products have been used as hard phases to modify silicone rubber by chemical bonding. Ardhyananta *et al.* prepared three types of polybenzoxazine–polysiloxane hybrids and inferred that the pendant group of polysiloxanes strongly affects and controls the thermal and mechanical properties.^9^ Zhang *et al.* reported PDMS-*g*-azobenzene dielectric elastomers with remarkably enhanced dielectric constants and high dielectric strengths, owing to the high dipole moments of azobenzenes.^10^ Magennis *et al.* devised a simple method to prepare silicone rubber-*g*-acrylates.^11^ Deshpande *et al.* researched the thermal degradation behaviors of PDMS, poly(dimethyldiphenylsiloxane) and poly(dimethyldiphenyl)siloxane copolymers and reported that the activation energies decreased on incorporation of phenyl groups.^12,13^ In recent years, biopolymers, based on renewable biomass feedstock, such as lignin, cellulose and rosin, have attracted more attention, due to the decline of fossil reserves and serious environmental pollution.^14–17^

Rosin, which is an abundantly and widely distributed renewable natural resin, is composed of 90% resin acid and 10% neutral compounds.^18,19^ Owing to its unique structure, which includes a large hydrogenated phenanthrene ring, rosin is similar to cyclic aliphatic or aromatic compounds in terms of molecular rigidity.^20^ On account of a carboxylic acid group and a conjugated carbon–carbon double bond as active functional groups, rosin acid can be modified through esterification, Diels–Alder addition, and amidation reactions.^20,21^ The obtained rosin-based products, which may serve as alternatives to petroleum-based cyclic aliphatic or aromatic monomers, can be introduced into polymers, such as epoxy resins and polyurethanes, and silicone rubber to impart excellent performance.^18,22,23^ Deng *et al.* prepared a series of novel rosin-based siloxane epoxy resins with better mechanical and thermal properties by increasing the cross-linking density and forming a protective residue.^24^ Xu *et al.* prepared a series of novel rosin-based, waterborne polyurethanes with good mechanical properties, thermal stability, and water resistance with a great potential for applications.^25^ In our previous study, a series of high temperature vulcanized silicone rubber containing side-chain rosin and its derivatives were prepared.^22,26,27^ The incorporation of rosin and its derivatives into silicone rubber systems remarkably improves the mechanical and thermal properties. However, to the best of our knowledge, modified RTV silicone rubber derivatized with rosin has seldom been studied.

**Fig. 1:**
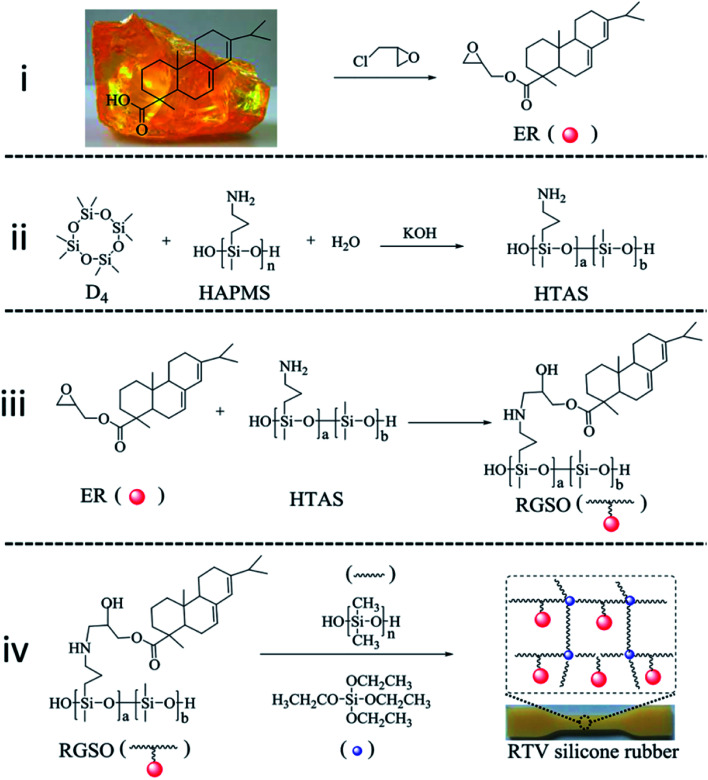
Route for the synthesis of RTV silicone rubber. (i) Synthetic route for ER. (ii) Synthetic route for HTAS. (iii) Synthetic route for RGSO. (iv) Synthetic route for RTV silicone rubber.

In this work, rosin-grafted polydimethylsiloxane (RGSO) was prepared *via* ring-opening reaction of epoxy and amino groups under relatively mild conditions (Fig. 1(iii)). The effects of glycidyl ester of rosin acid (ER) on the relative molecular weight and rheological properties of RGSO were investigated. PDMS was grafted with rosin, followed by condensation reaction with PDMS matrix, to obtain rosin-modified RTV silicone rubber (Fig. 1(iv)). The effects of ER on the morphology, thermal properties, mechanical properties, and dynamic mechanical properties of RTV silicone rubber were all explored.

## Materials and methods

### Materials

Purified rosin (*ca.* ≧ 95%) was supplied by Hunan Pine Forest Technologies Co. Ltd. Hydroxy-terminated PDMS (5000 MPa s) was purchased from Hubei New Universal Chemical Co. Ltd. Octamethylcyclotetrasiloxane (D_4_) and 3-aminopropyl(diethoxy)methylsilane were purchased from Wanda Chemical Co. Ltd. Tetraethoxysilane (TEOS), potassium hydroxide (KOH), toluene, sodium hydroxide, calcium oxide, acetic acid, celite, epichlorohydrin, benzyltriethylammonium chloride and dibutyltin dilaurate were supplied by Aladdin Chemical Reagent Co. Ltd. All chemicals were used without further purification.

### Synthesis of ER

ER with an epoxide equivalent weight of 352 g mol^−1^ (theoretical 358 g mol^−1^) was prepared by the reaction of rosin and epichlorohydrin, according to a literature procedure.^28^ Rosin (50.0 g), epichlorohydrin (157.3 g) and benzyltriethylammonium chloride (0.381 g) were charged into a 500 mL flask. The mixture was maintained at 117 °C for 2 h under nitrogen atmosphere and then cooled to 60 °C. Sodium hydroxide (6.62 g) and calcium oxide (9.272 g) were added to the flask and the mixture was held at around 60 °C for another 3 h. The mixture was filtered through celite and filter paper. The target product was obtained after distillation of the filtrate under vacuum at 100 °C to remove excess epichlorohydrin (Fig. 1(i)).

### Synthesis of hydrolyzate of 3-aminopropyl(diethoxy)methylsilane (HAPMS)

HAPMS was obtained by hydrolyzation of 3-aminopropyl(diethoxy)methylsilane, according to a literature procedure.^22^ A flask was filled with equal volumes of water and 3-aminopropyl(diethoxy)methylsilane. The mixture was maintained at 80 °C for 4 h. The target product was obtained after removal of excess water under vacuum at 100 °C.

### Synthesis of hydroxy-terminated amino polydimethylsiloxane (HTAS)

A 1000 mL flask, equipped with a stirrer, thermometer and condenser, was charged with HAPMS (50 g), D_4_ (450 g), KOH aqueous solution (10 wt%, 2000 μL) and a certain amount of water under nitrogen atmosphere. The mixture was heated at 140 °C for 6 h, after which it turned into a transparent and viscous liquid. After the mixture was cooled to 60 °C, aqueous acetic acid (10 wt%, 2143 μL) was added. The volatiles were removed under vacuum at high temperature. Finally, HTAS (450 g, 90% yield, 5000 MPa s, Fig. 1(ii)) with an amino content of 0.84 mmol g^−1^ (theoretical 0.85 mmol g^−1^) was obtained.

### Preparation of RGSO

HTAS (10 g) and ER (1.5 g, 3 g, 4.5 g or 6 g) were charged into a flask equipped with a stirrer, inert gas inlet, thermometer, and reflux condenser, and the mixture was heated at 80 °C for about 1 h. The molar ratios of the amino groups and epoxide groups were 1 : 0.5, 1 : 1, 1 : 1.5 and 1 : 2, respectively (Table S3†). The RGSO products obtained were designated as RGSO-1, RGSO-2, RGSO-3, and RGSO-4, respectively (Fig. 1(iii)).

### Preparation of modified RTV silicone rubber

To a flask were added the products RGSO-1, RGSO-2, RGSO-3, or RGSO-4, obtained as described above, and PDMS (20 g) under vigorous stirring and dry nitrogen. Then the crosslinking agent TEOS (4.5 g) and dibutyltin dilaurate catalyst (100 μL) were added and mixed homogeneously. Finally, the mixture was quickly poured into a mould and cast after removing the air bubbles. The RTV silicone rubber sheets with smooth surfaces were obtained after curing for 7 days at room temperature (Fig. 1(iv)). They were designated correspondingly as SRER-2, SRER-3, SRER-4, and SRER-5. As a reference material, the RTV silicone rubber using PDMS matrix as the primary polymer was also prepared under the same conditions (Table S1, ESI†).

### Characterizations and measurements

#### NMR

^1^H-NMR and ^13^C-NMR spectra were obtained at 40 °C with an AV400 spectrometer (Bruker, Germany) at frequencies of 400.13 and 100.61 MHz, respectively. Deuterated chloroform (CDCl_3_) was used as the solvent and tetramethylsilane (TMS) as an internal standard. The chemical shifts were referenced to the signals of CDCl_3_ and TMS.

#### Gel permeation chromatography (GPC)

Molecular weights and molecular weight distributions in polymers were determined by GPC using a Waters Breeze system (Waters, America), equipped with a Waters 2414 detector. The eluent was HPLC-grade THF and the flow rate was 1 mL min^−1^.

#### Rheology

Rheological studies of RGSO were conducted with a Haake Mars II rotational rheometer (Haake, Germany) under steady shear flow at 25 °C. Apparent viscosities were obtained at given shear rates ranging from 1 to 100 s^−1^.

#### Density

The densities of samples were measured using a pycnometer.

#### Hardness

The hardnesses of samples were measured using an LX-A durometer (Eide fort, China) at 23 °C and approx. 50% relative humidity (RH).

#### Thermogravimetric analysis (TG)

Thermal stabilities of the samples were determined with a TG209F1 (Netzsch, Germany). The samples were heated from 25 °C to 800 °C at a rate of 10 °C min^−1^ under nitrogen atmosphere.

#### Mechanical properties

Dumbbell-shaped specimens were prepared and tested for mechanical performance using a capacity of 500 N with a UTM6502 universal testing machine (Suns, China). The samples were subjected to a cross-head speed of 500 mm min^−1^ at 23 °C and approx. 50% RH and an average of five measurements was reported.

#### Scanning electron microscopy (SEM)

Sections of silicone rubber were sputter-coated with gold. Then their morphologies were studied using a QUANTA 200 (FEI, Holland) scanning electron microscope at a voltage of 10 kV.

#### Dynamic mechanical analysis (DMA)

The dynamic mechanical analyses of the samples were conducted using a DMA Q800 (TA Instruments, USA). The analyses were carried out at a frequency of 1 Hz from −135 °C to −75 °C and a heating rate of 3 °C min^−1^ by stretching mode.

#### Crosslinking density

The crosslinking density of RTV silicone rubber was determined by equilibrium swelling method. RTV silicone rubber (about 0.2 g) and toluene (25 mL) was placed in a sealed vessel at 25 °C. After immersing in toluene for 48 h, the samples were blotted with filter paper to remove excess toluene and weighed. Then, they were again immersed in toluene. This process was repeated every 3 h, until the weight remained constant, which indicated that the equilibrium swelling was achieved. The crosslinking density was calculated using the following eqn (1) and (2):^29–31^1
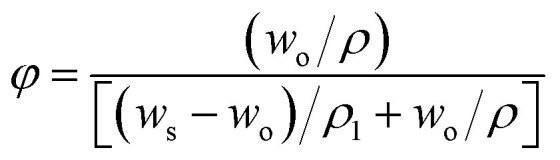

2 *γ*_e_ = *ρ*/*M*_C_ = −[ln(1 − *φ*) + *φ* + *X*_1_*φ*^2^]/(*v*_o_*φ*^1/3^)

In these equations, *φ* is the volume fraction, *w*_o_ is the weight of the original sample, *ρ* is the density of RTV silicone rubber before swelling, *w*_s_ is the weight of swollen RTV silicone rubber, *ρ*_1_ is the density of toluene whose value is 0.87 g cm^−3^, *γ*_e_ is the crosslinking density, *M*_C_ is the average molecular weight between the crosslinking points, *X*_1_ is the interaction parameter of polymer and solvent whose value is 0.465, and *v*_o_ is the molar volume of toluene whose value is 106.54 × 10^−3^ L mol^−1^.

#### The amino content of HTAS

The amino content of HTAS was determined as follows. HTAS (1.0 g), toluene (20 mL) and carbon tetrachloride (10 mL) were mixed in an Erlenmeyer flask. Crystal violet was used as an indicator. The mixture was titrated using standard solution of perchloric acid-acetic acid (0.1 mol L^−1^) until the mixture changed to yellow. A control experiment without HTAS was also used as a blank reference. The formula for calculating the amino value is as follows:3
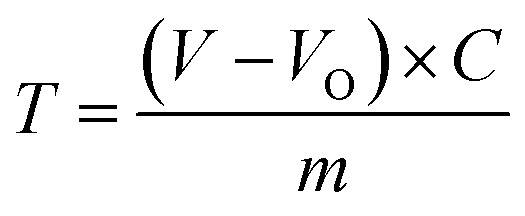
where *V* is the consumption of the perchloric acid solution by the test sample (mL); *V*_O_ is the consumption of the perchloric acid solution by the blank experiment (mL); *C* is the concentration of the perchloric acid solution (mol L^−1^); *m* is the quantity of the sample (g).

## Results and discussion

### Characterization of RGSO

#### NMR analysis

**Fig. 2:**
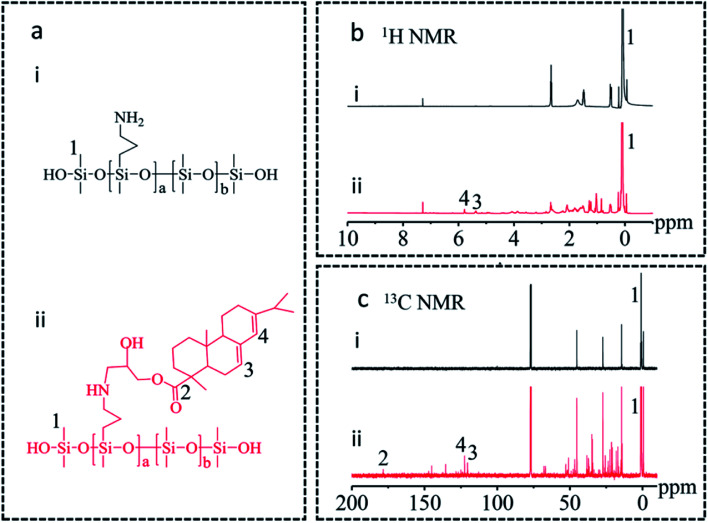
NMR spectroscopic analysis. (a) (i) Molecular formula of HTAS. (ii) Molecular formula of RGSO. (b) (i) ^1^H NMR spectrum of HTAS. (ii) ^1^H NMR spectrum of RGSO. (c) (i) ^13^C NMR spectrum of HTAS. (ii) ^13^C NMR spectrum of RGSO.

The structures of HTAS and RGSO were confirmed by ^1^H NMR and ^13^C NMR spectroscopy, as shown in Fig. 2. The structures of HTAS and RGSO are shown in Fig. 2a. Fig. 2b(ii) shows the ^1^H NMR spectrum of RGSO, in which the characteristic peaks at 5.77 and 5.37 ppm corresponded to the protons on unsaturated carbons and the peak at about 0.14 ppm was attributed to the silicon methyl.^22^ In ^13^C NMR spectra, the signal at about 0.05 ppm was assigned to silicon methyl. Compared to spectrum 2ci, spectrum 2cii showed the characteristic peak for ester at 178 ppm and the peaks for carbon–carbon double bond at 120 ppm and 123 ppm.^22^

#### GPC analysis

**Fig. 3:**
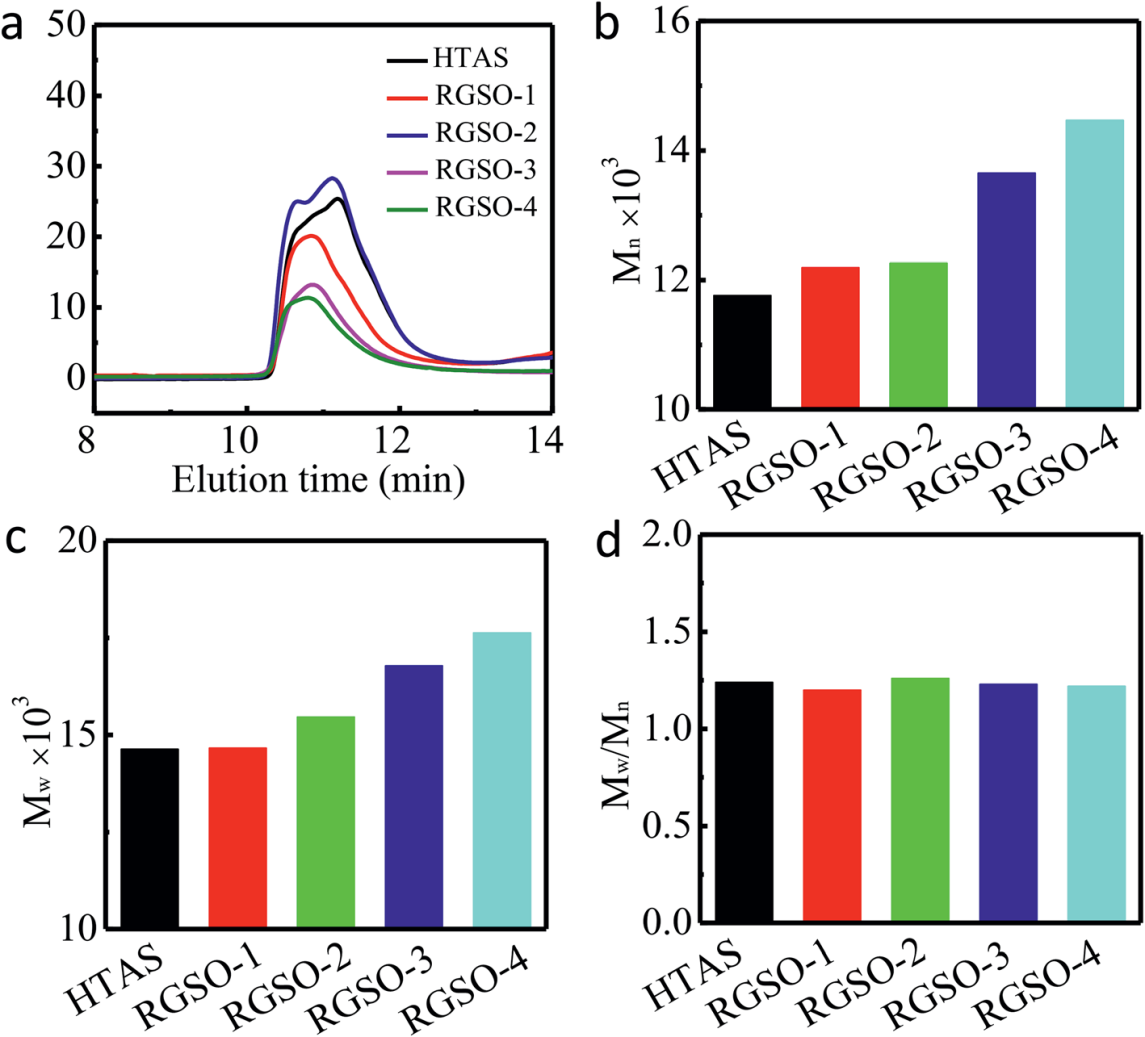
GPC analyses of HTAS and RGSO. (a) GPC curves of HTAS and RGSO. (b) Number average molecular weights (*M*_n_) of HTAS and RGSO. (c) Weight average molecular weights (*M*_w_) of HTAS and RGSO. (d) Polydispersity indexes of HTAS and RGSO.

The molecular weights of HTAS and RGSO were determined by GPC as shown in Fig. 3a. The number average molecular weights (*M*_n_) of HTAS, RGSO-1, RGSO-2, RGSO-3, and RGSO-4 were 11 765, 12 195, 12 262, 13 654, and 14 472 Da, respectively (Fig. 3b). The weight average molecular weights (*M*_w_) of HTAS, RGSO-1, RGSO-2, RGSO-3, and RGSO-4 were 14 635, 14 660, 15 462, 16 787, and 17 638 Da, respectively, with low dispersities of 1.24, 1.20, 1.26, 1.23, and 1.22, respectively (Fig. 3c and d). The GPC curves for the number average molecular weight (*M*_n_) of HTAS shifted clearly towards higher molecular weights of RGSO because of the chain extension. These results further support the successful grafting of ER onto the side chains of HTAS.^20^

#### Rheological properties

**Fig. 4:**
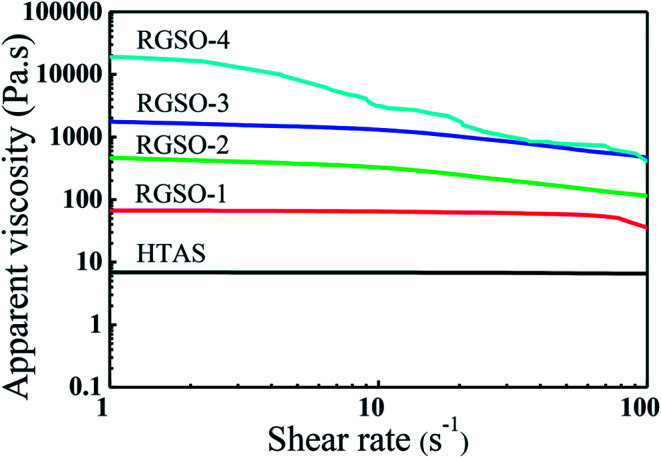
Changes in apparent viscosities *versus* shear rates of HTAS and RGSO.

The steady shear plots of HTAS and RGSO with different mass fraction of ER are shown in Fig. 4. The apparent viscosity increased from 7 Pa s (HTAS) to 18 980 Pa s (RGSO-4) with an increase in grafting of ER. Since the introduction of ER increased the degree of branching in the polysiloxane molecules, which was also consistent with the GPC results, the intermolecular entanglement of chain segments also increased.^32^ Moreover, compared to the Newtonian property of HTAS, the apparent viscosity of RGSO decreased with an increase in shear rate from 1 to 100 s^−1^, exhibiting a pseudoplastic property. Due to the separation of the winding structure, under the action of shearing force, it was aligned in the flow direction with increasing shear rate.^33^

#### Characterization of RTV silicone rubber

Modified RTV silicone rubber, based on PDMS and RGSO, was synthesized *via* a condensation reaction in humid environment at room temperature. The crosslinking process was affected by all the components and also the experimental conditions. In our experiments, a certain amount of dibutyltin dilaurate catalyst (100 μL), PDMS (5000 MPa s, 20 g), HTAS (5000 MPa s, 10 g) and TEOS crosslinking agent (4.5 g) were used to prepare modified silicone rubber under the same conditions. To investigate the effect of ER on the morphologies and thermal and mechanical properties of RTV silicone rubber, 1.5, 3.0, 4.5, and 6.0 g of ER was added to obtain 5, 10, 15 and 20 wt%, respectively, of ER-containing RTV silicone rubber. These products were designated as SRER-2, SRER-3, SRER-4, and SRER-5, respectively. As a reference material, PDMS matrix as primary polymer with 7% TEOS was used to prepare RTV silicone rubber because of its relatively good mechanical properties (Table S2, ESI†). And the unmodified silicone rubber was designated as SRER-1. The compositions of RTV silicone rubbers are listed in Table 1.

**Table 1:** The compositions of RTV silicone rubbers

Sample	PDMS (g)	HTAS (g)	ER (g)	TEOS (g)	Catalyst (μL)	ER (wt%)
SRER-1	30	0	0	2.1	100	0
SRER-2	20	10	1.5	4.5	100	5
SRER-3	20	10	3.0	4.5	100	10
SRER-4	20	10	4.5	4.5	100	15
SRER-5	20	10	6.0	4.5	100	20

#### Morphologies

**Fig. 5:**
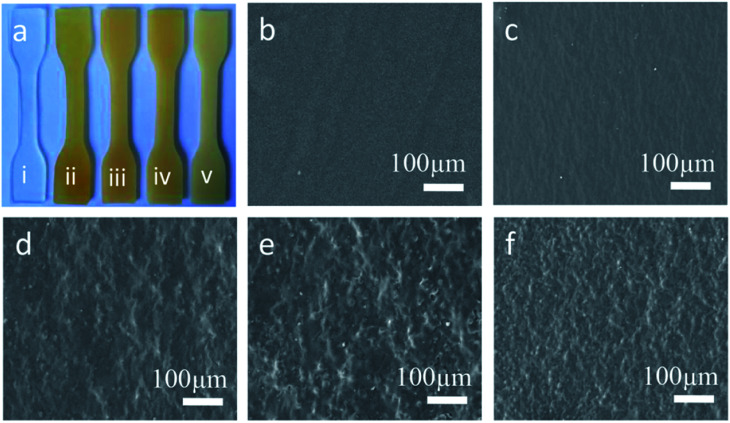
Morphologies and microstructures of RTV silicone rubbers. (a) Photo of (i). SRER-1, (ii). SRER-2, (iii). SRER-3, (iv). SRER-4, (v). SRER-5. SEM images of (b) SRER-1, (c) SRER-2, (d) SRER-3, (e) SRER-4, (f) SRER-5.

The surface morphologies and microstructures of RTV silicone rubbers are shown in Fig. 5. Unmodified RTV silicone rubber without grafted ER appears clear and colorless (Fig. 5a(i)). However, the ER-modified silicone rubbers appear opaque and darker (Fig. 5a(ii–v)), due to changes in microstructure. The morphologies of the RTV silicone rubbers were investigated by SEM, as shown in Fig. 5b–f. Compared to the unmodified RTV silicone rubber, the surface microstructure of the ER-modified silicone rubber appears rougher with the increase in ER content. This can be attributed to the fact that ER with a bulky hydrogenated phenanthrene ring has stronger polarity and rigidity than the polysiloxane and tends to aggregate in silicone rubber.^22^ Microphasic separation occurred between ER and polysiloxane in the silicone rubber, with increase in the bulk of hydrogenated phenanthrene ring of ER.^34^ So the SEM image of SRER-5 showed a relatively rougher surface and a pronounced microphasic separation. Moreover, the surface morphology and microstructure of RTV silicone rubber in Fig. 5 showed uniform distribution of ER in polysiloxane, which is consistent with the TEM result (Fig. S3†).

#### Mechanical properties

**Fig. 6:**
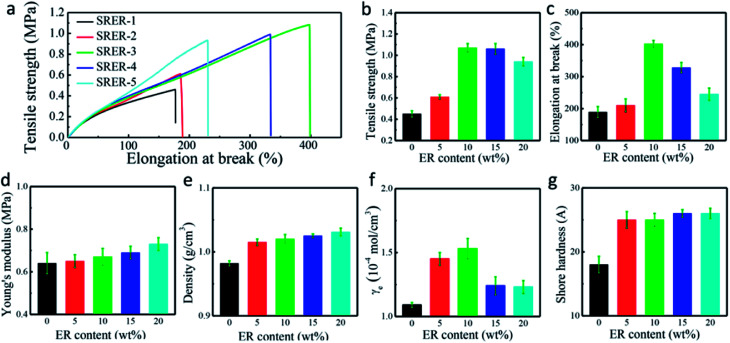
Mechanical properties of RTV silicone rubbers with different ER contents. (a) Stress–strain curves of RTV silicone rubbers. (b) Tensile strengths of RTV silicone rubbers. (c) Elongation at break of RTV silicone rubbers. (d) Young's moduli of RTV silicone rubbers. (e) Densities of RTV silicone rubbers. (f) Crosslinking densities of RTV silicone rubbers. (g) Shore hardnesses of RTV silicone rubbers.

As illustrated in Fig. 6, the mechanical properties of RTV silicone rubber grafted with different amounts of ER were determined using a universal testing machine. The stress-strain curves of RTV silicone rubber (Fig. 6a) and the test for mechanical properties of SRER-1 and SRER-3 (Fig. S1, ESI†) suggested that ER-modified RTV silicone rubber possessed better mechanical properties. Compared with the SRER-1 sample, the tensile strength and elongation at break of the modified RTV silicone rubber were remarkably enhanced (Fig. 6b and c). The tensile strength and the elongation at break of SRER-3 were 1.07 MPa and 402%, respectively, which showed an increase of 138% and 113%, respectively. The Young's modulus of RTV silicone rubber also showed a slight improvement from 0.64 MPa to 0.73 MPa (Fig. 6d).

**Fig. 7:**
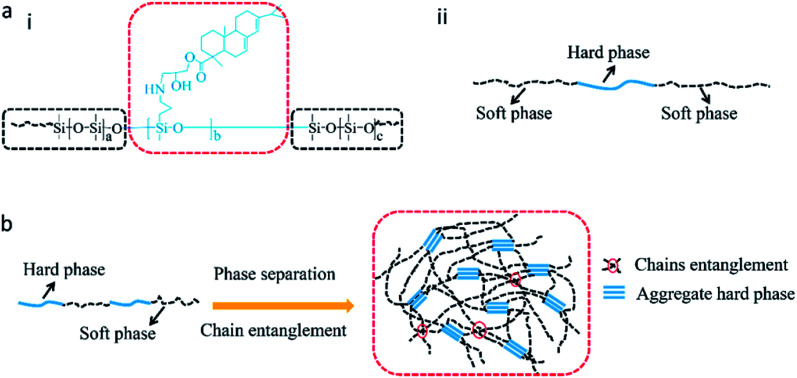
Schematic illustration of the influence on mechanical properties of silicone rubber. (a) (i) Molecular structure of RGSO. (ii) Hard phase and soft phase in RGSO. (b) Microphasic separation and chain entanglement in network structure of silicone rubber.

Improvements in tensile strength, elongation at break, and Young's modulus of the modified RTV silicone rubber were observed owing to the chemical incorporation of hydrogenated phenanthrene ring of ER into polysiloxane.^35^ A hard phase is mainly composed of ER-grafted polysiloxane and a soft phase contains polysiloxane chains (Fig. 7a). The density of hard phase increases with increasing amount of ER-grafted polysiloxane. This leads to an increase in cohesion energy of silicone rubber.^22^ ER on the side chains of polysiloxane could increase sufficiently the chain entanglement between rosin and polysiloxane chains to achieve good mechanical properties (Fig. 7b).^20^ Crosslink density is an important factor for the remarkable reinforcement of silicone rubber.^36^ The crosslinking density increased slightly with an increase in grafted ER from 1.09 × 10^−4^ mol cm^−3^ to 1.53 × 10^−4^ mol cm^−3^ (Fig. 6f). The mechanical properties of silicone rubber increased obviously due to the synergistic effects of increases in density of hard phase and crosslinking density (Fig. 7b). However, when the amount of grafted ER exceeds 10 wt%, the tensile strength and elongation at break of the ER-modified RTV silicone rubber decreased slightly with the decrease in crosslinking densities in SRER-4 and SRER-5. As the amount of grafted ER exceeded 10 wt%, ER-rich domains were formed in the RTV silicone rubber, because of the more polar and rigid ER. A marked microphasic separation occurred between ER-rich domains and polysiloxane in the structure of silicone rubber, which was evident in the SEM images (Fig. 5e and f).^18^ Decrease in crosslinking density and a marked microphasic separation could seriously affect the mechanical properties of silicone rubber. Moreover, the density and the Shore hardness increased with increase in ER loading from 0.982 to 1.031 g cm^−3^ and from 18 to 26 A, respectively (Fig. 6e and g). Therefore, the effective improvement in the mechanical properties of RTV silicone rubber could be attributed to the hard phase of ER, the degree of chain entanglements, and also the uniform distribution of ER in silicone rubber.

#### Thermal properties

**Fig. 8:**
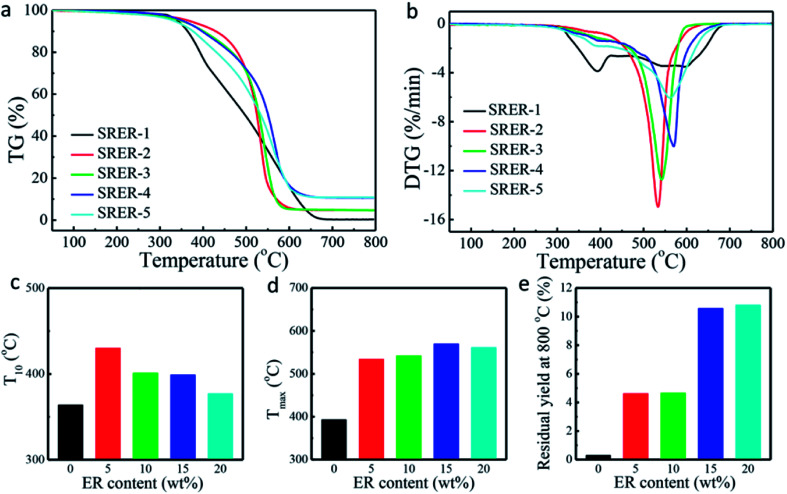
Thermal stabilities of RTV silicone rubbers. (a) TG curves of RTV silicone rubbers. (b) DTG curves of RTV silicone rubbers. (c) Temperatures corresponding to 10% weight loss of RTV silicone rubbers. (d) Temperatures of maximum rate of weight loss of RTV silicone rubbers. (e) Residual yields at 800 °C of RTV silicone rubbers.

Thermal degradation of RTV silicone rubber was investigated by TG, as shown in Fig. 8. The TG and DTG curves of RTV silicone rubber are shown in Fig. 8a and b, suggesting that ER-modified RTV silicone rubber was more stable than unmodified RTV silicone rubber. When 5 wt% ER was grafted onto the side chains of RTV silicone rubber, the temperature corresponding to 10% weight loss increased from 364 °C (SRER-1) to 430 °C (SRER-2) and it decreased with increasing ER content. Since the C–N bond energy of amines is relatively low, such a bond breaks first at a specific temperature (Fig. 8c).^37^ Furthermore, the temperatures corresponding to 10% weight loss of modified silicone rubbers were significantly higher than that of SRER-1. This was due to the chemical introduction of ER containing a phenanthrene ring into the RTV silicone rubber and the subsequent increase in crosslinking density (Fig. 6f). In previous studies, a cyclization of Si–O bonds resulted in the formation of volatile cyclic products in the presence of incompletely reacted hydroxyl-containing impurities at relatively low temperature. This led to poor thermal stability of unmodified silicone rubber.^38^ Grafting of ER onto silicone rubber increased the degree of chain entanglements in SRER silicone rubber along with the crosslinking density and the hard phase density, which inhibited the molecular motion of polysiloxane chains.^20^ This prevented the rearrangement of Si–O bonds of polysiloxane and the formation of cyclic oligomers arranged in the order D_3_ to D_13_ and decreased the rate of thermal degradation (Fig. 8d).^39,40^

The temperature corresponding to maximum rate of weight loss was also delayed by 177 °C from 393 °C (SRER-1) to 570 °C (SRER-4). Owing to the obvious polarity of the Si–O bond and flexibility of the -(Si–O)_*n*_-segments, the traces of residual hydroxyl groups could themselves accelerate the decomposition, leading to rapid degradation at 393 °C (SRER-1).^39^ However, the ER-modified silicone rubber was prevented from degrading to cyclic oligomers, because of the restricted mobility of polysiloxane chains, which prevented the rearrangement of Si–O bonds in polysiloxane.^41^ As a result, the decomposition temperature of modified silicone rubber (SRER-4) was delayed up to 570 °C. At these higher temperatures, the obvious increase in mobility of the chain and molecular motion facilitated random degradation.^38^ Ultimately the unmodified silicone rubber SRER-1 with 0.30% residue at 800 °C was almost entirely degraded into cyclic oligomers. The ER-modified silicone rubber had more residual yield compared to the unmodified silicone rubber SRER-1 (Fig. 8e).^42^

#### Dynamic mechanical properties

**Fig. 9:**
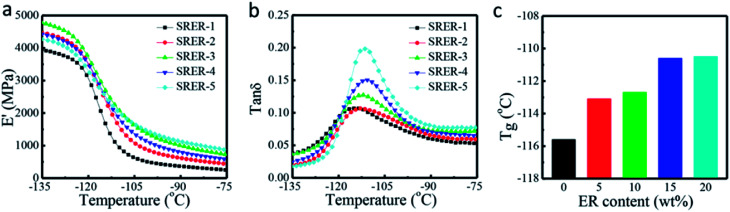
Dynamic mechanical properties of silicone rubbers. (a) Curves for storage moduli (*E*′) of RTV silicone rubbers. (b) Tan *δ* curves of RTV silicone rubbers. (c) *T*_g_ values of RTV silicone rubbers.

In Fig. 9a, the *E*′ curves for rosin-modified RTV silicone rubbers are all higher than that of unmodified RTV silicone rubber, suggesting the former had more rigid molecular structures than the latter. This rigidity could be attributed to the bulky hydrogenated phenanthrene ring in ER, which increased the hard phase density. Moreover, it decreased the segmental mobility between the hard phase and soft phase by restricting on the molecular motion.^20^ Compared to SRER-3, the *E*′ values of SRER-4 and SRER-5 decreased slightly, due to the aggregation of ER and the decrease in crosslink density.^38^

The peak temperature of tan *δ* corresponded to the glass transition temperature (*T*_g_). Fig. 9b shows that the tan *δ* curves of RTV silicone rubber grafted with different weight ratios of ER had only one distinct *T*_g_ from −135 °C to −75 °C. The *T*_g_ of RTV silicone rubber increased slightly from −115.6 °C to −110.5 °C (Fig. 9c) with an increase in ER loading. This was because of the more rigid hydrogenated phenanthrene ring structure of ER in modified RTV silicone rubber, which restricted the movement of the chains by increasing the rigidity of molecular chains and density due to chain entanglements.^43–45^

## Conclusion

In summary, RGSO was prepared *via* ring-opening reaction and was characterized. The relative molecular weight and viscosity of RGSO increased with increase in ER content. Then, the mixtures of RGSO and PDMS matrix, as primary polymers, were used to prepare rosin-modified RTV silicone rubber by condensation reaction at room temperature. Compared to unmodified silicone rubber SRER-1, rosin significantly enhanced the thermal stabilities and mechanical properties of rosin-modified RTV silicone rubbers. These results could be attributed to the strong rigidity and polar hydrogenated phenanthrene ring structure of ER, the increase in chain entanglement of polysiloxane and the perfect distribution of ER in the rosin-modified RTV silicone rubber. This study provides a strategy for sustainable development and exploration of biomass polymer-containing rosin-modified silicone rubber with superior performance.

## Funding

The authors express their gratitude for the financial support from National Natural Science Foundation of China (31570562); the Key Laboratory of Biomass Energy and Materials of Jiangsu Province of China (JSBEM-S-201504).

## Conflicts of interest

The authors declare no competing financial interest.

## Supplementary Material
